# Supplementary material for: Transcriptomic Insights into Mechanisms of Early Seed Maturation in the Garden Pea (Pisum sativum L.)
Source: Cells. 2020 Mar 23;9(3):779. doi: 10.3390/cells9030779 (PMC7140803; doi:10.3390/cells9030779)

Module correlations (WTO algorithm)

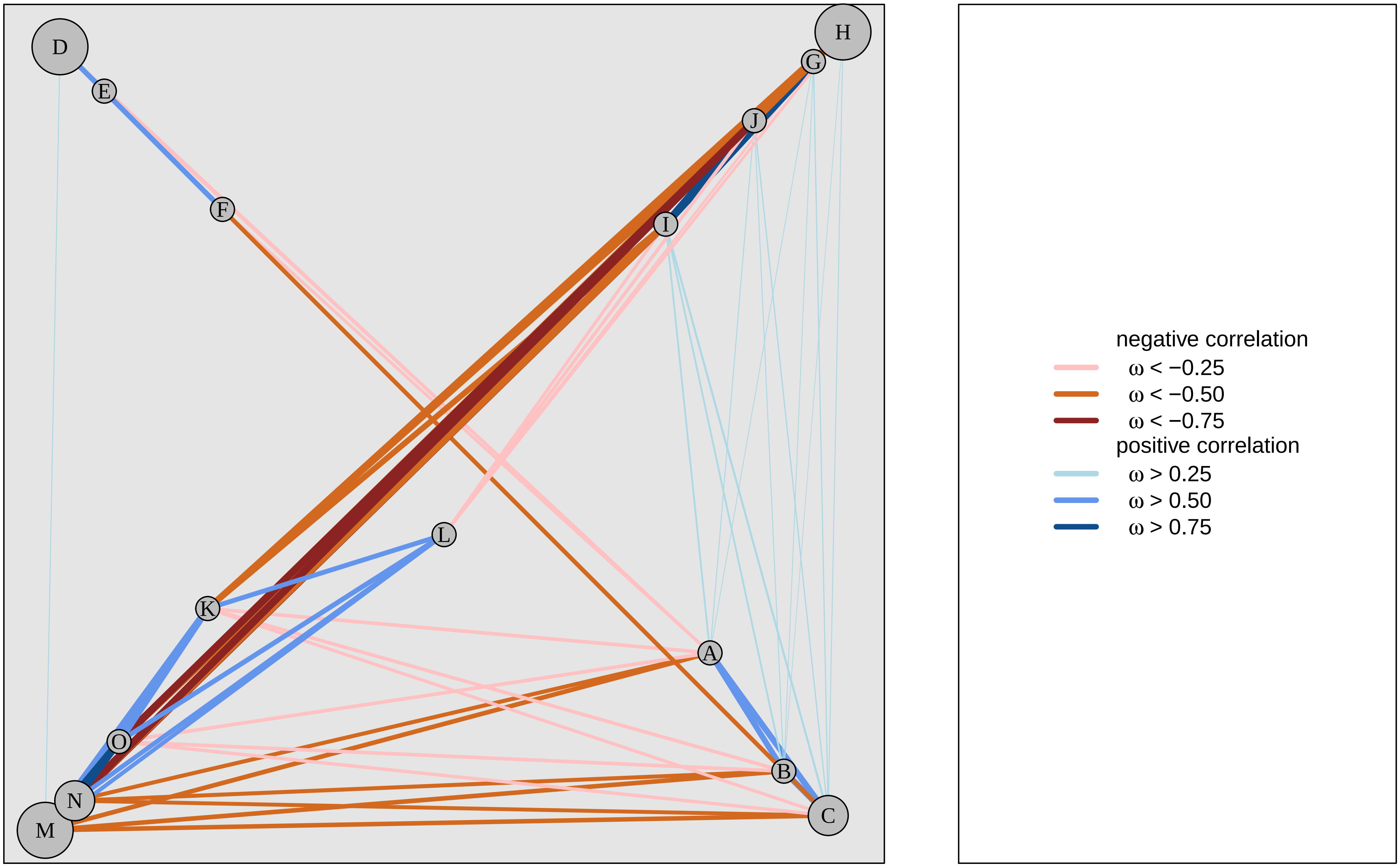

Module implications (basket algorithm)

all samples

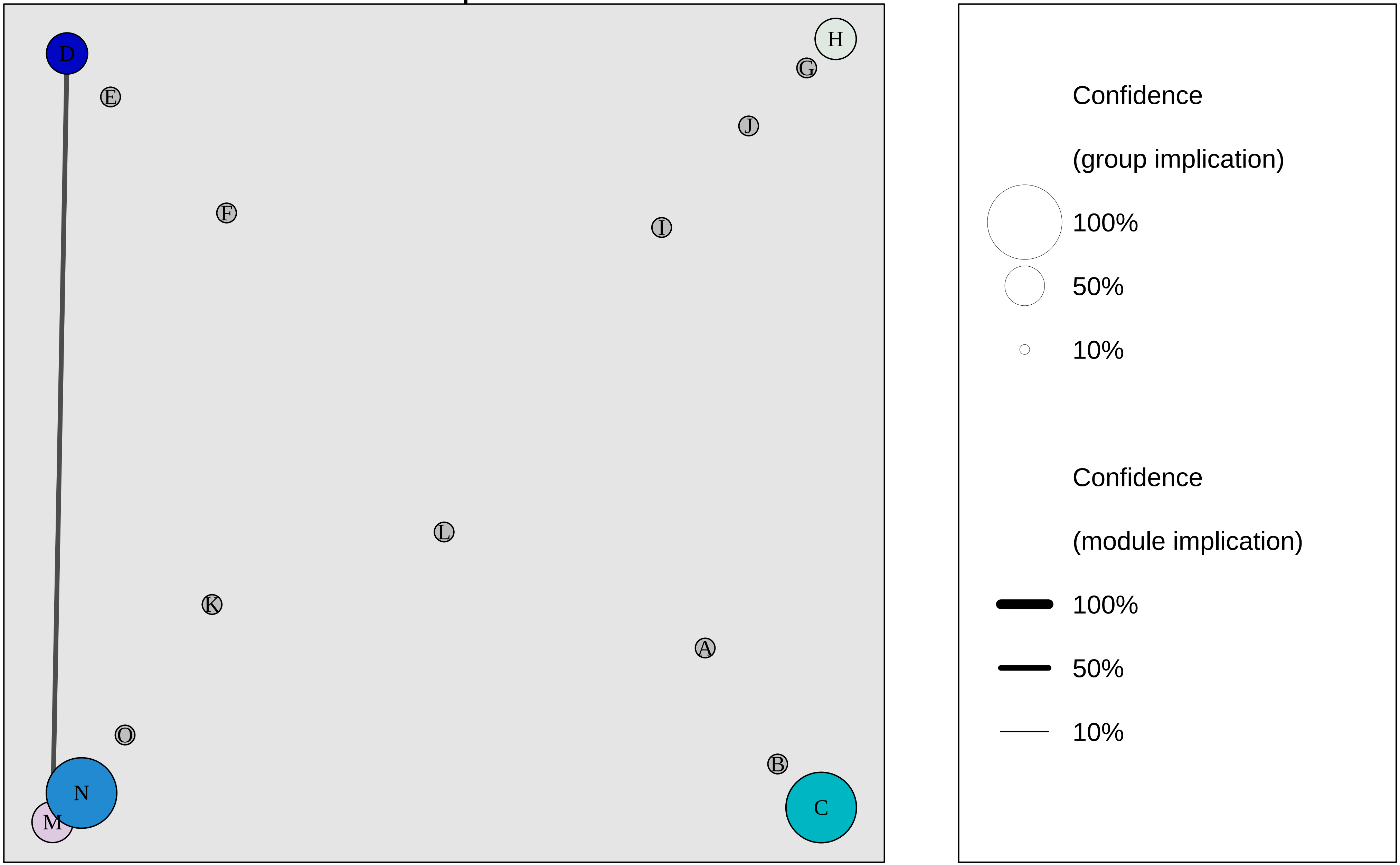

Module implications (basket algorithm)

Group samples: Zhewan−1(10)

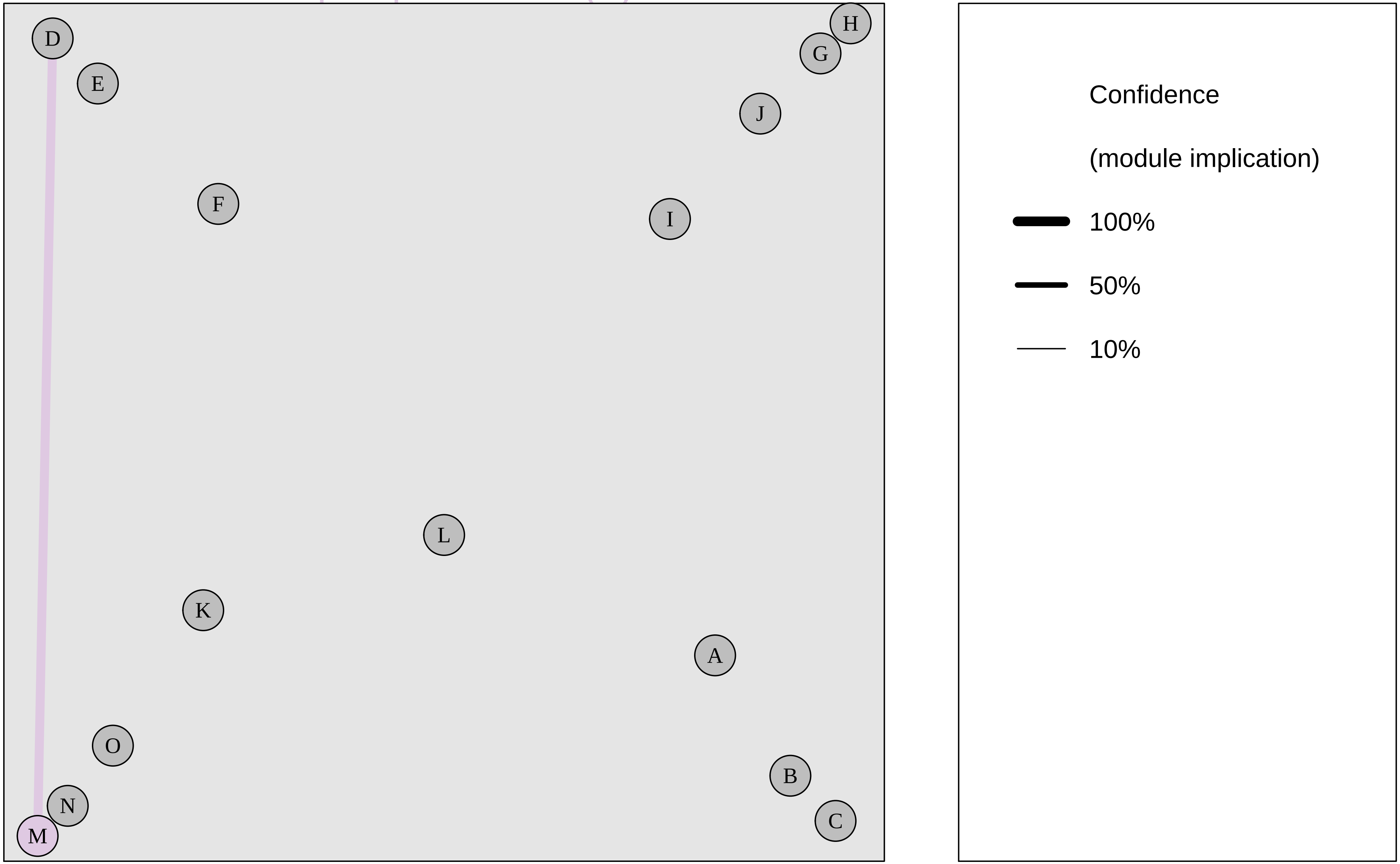

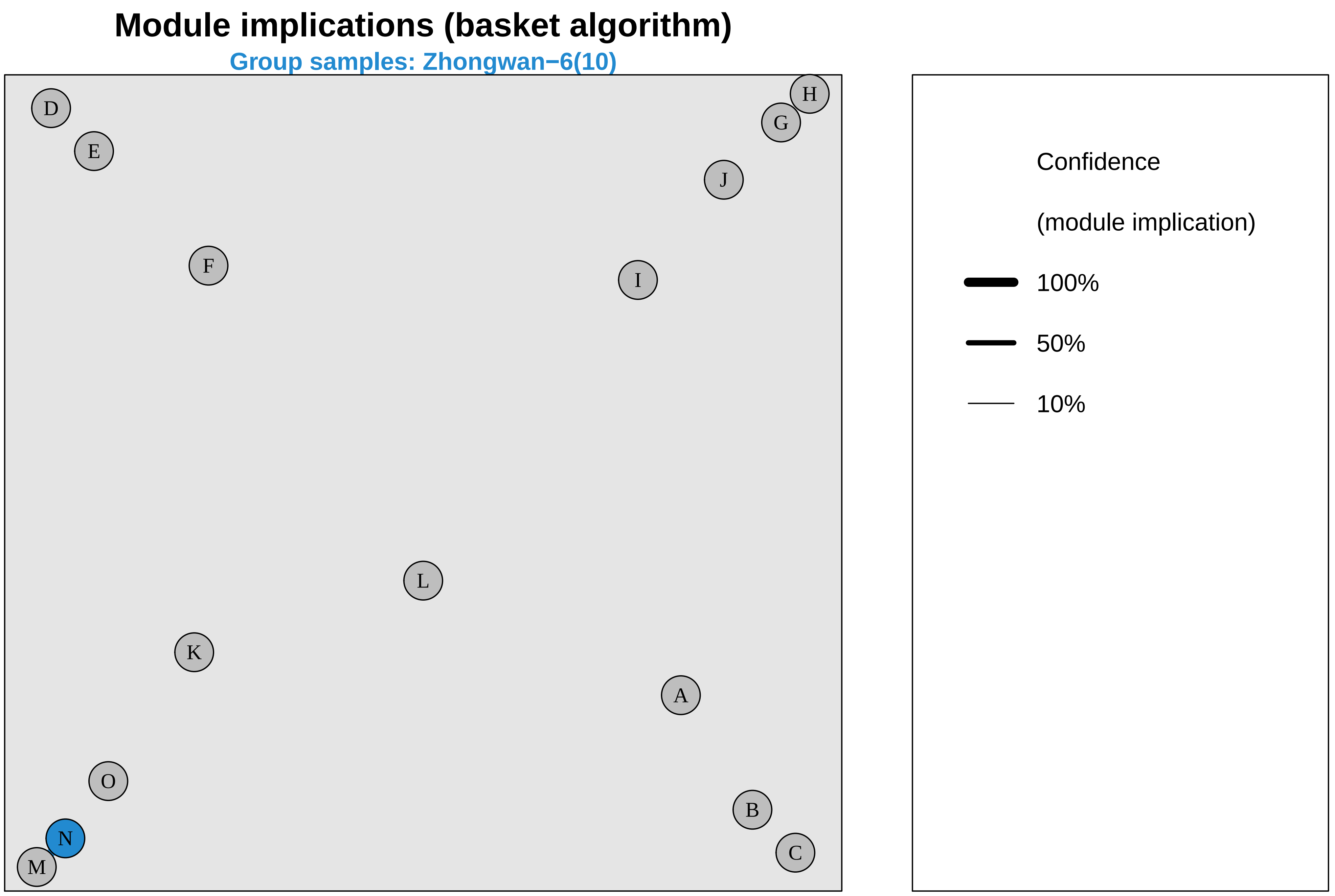
Module implications (basket algorithm)
Group samples: Zhongwan−6(10)
D
E
F
H
G
J
I
L
K
A
O
N
M
B
C
Confidence
(module implication)
100%
50%
10%

Group associations of the modules

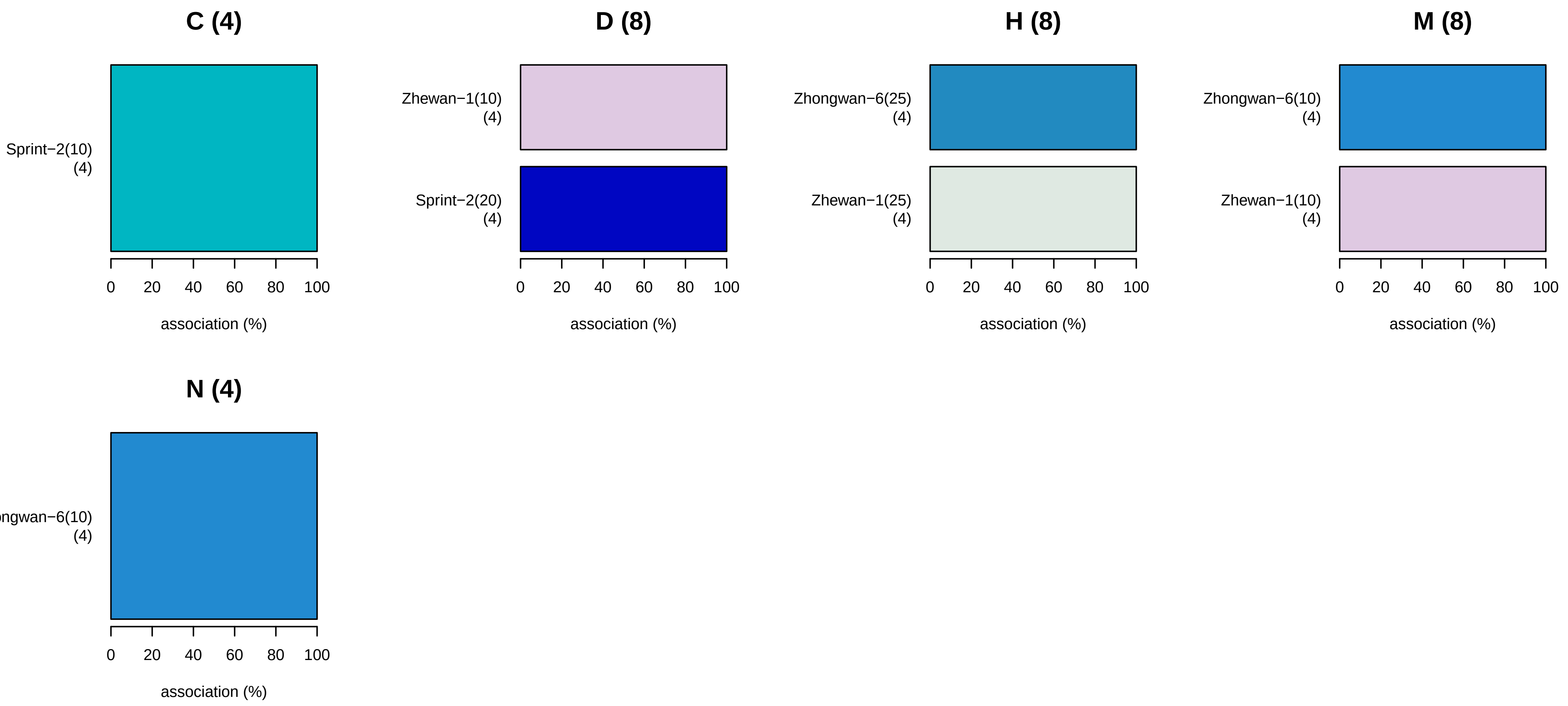

Module implications of the groups (basket algorithm)

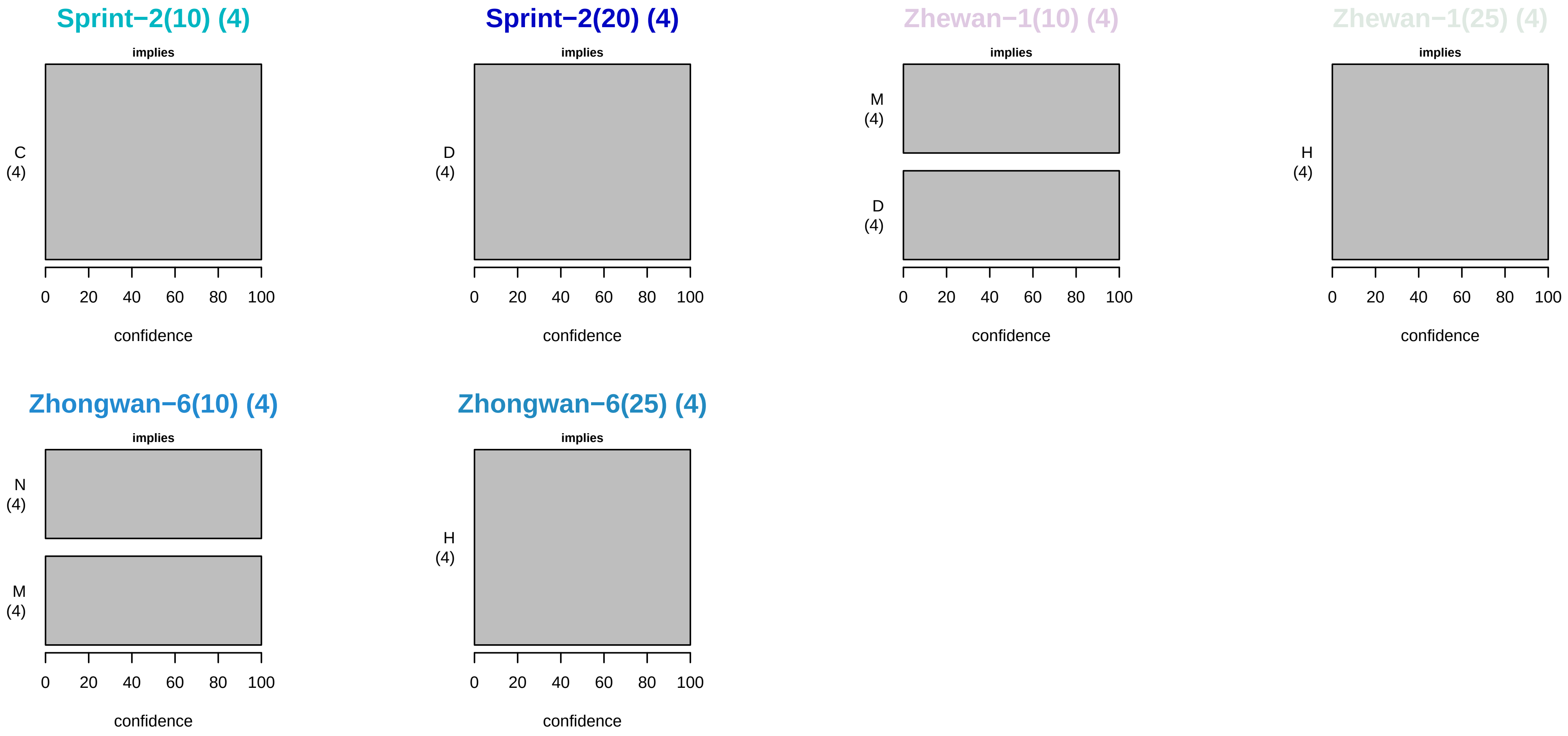

Group implications of the modules (basket algorithm)

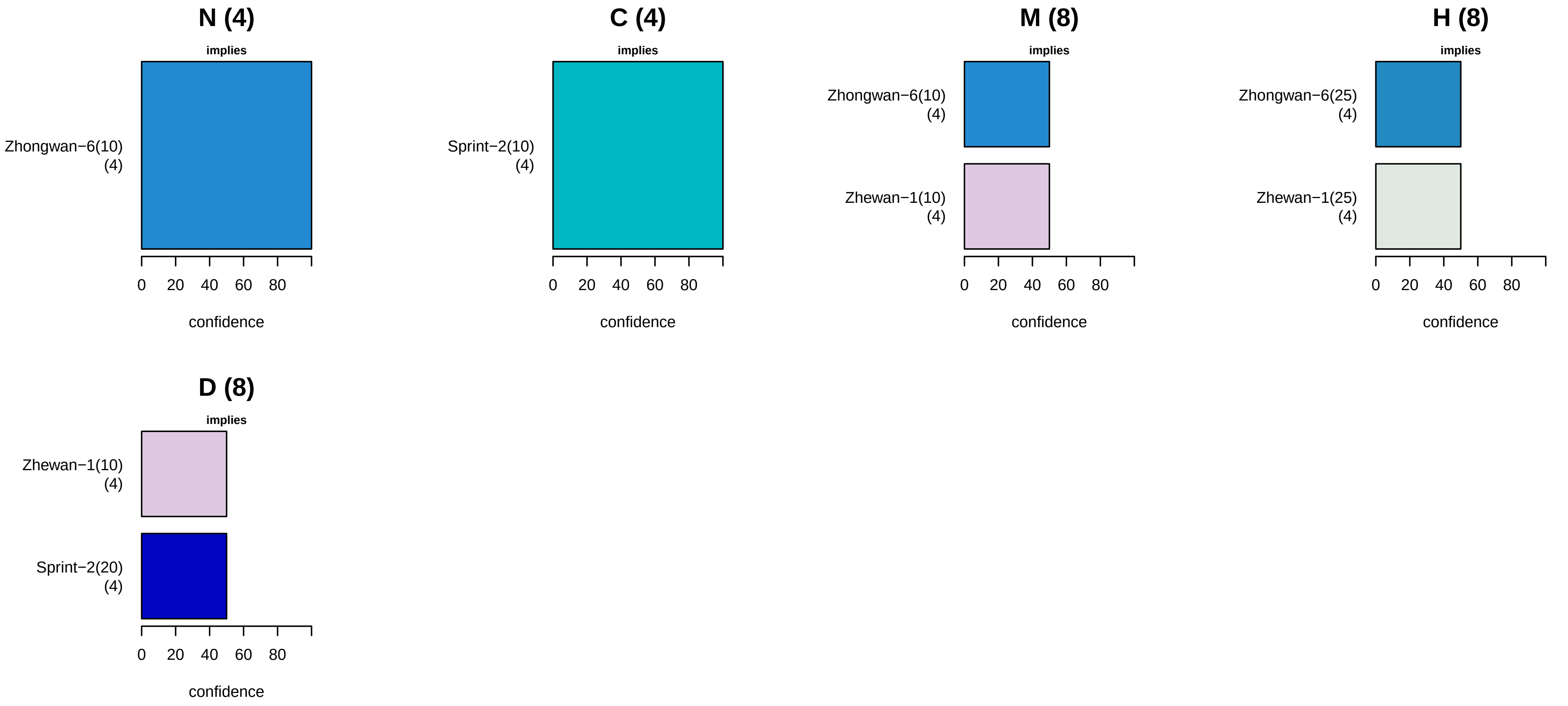

Supplement: Supplementary file 1 [file cells-09-00779-s001.zip › Supplementary materials/FileS4.zip!/FileS3/TFs - Results/Summary Sheets - Modules/K-Means Cluster/Relations.pdf]
